# Classification of the Universe of Immune Epitope Literature: Representation and Knowledge Gaps

**DOI:** 10.1371/journal.pone.0006948

**Published:** 2009-09-14

**Authors:** Vince Davies, Kerrie Vaughan, Rohini Damle, Bjoern Peters, Alessandro Sette

**Affiliations:** La Jolla Institute for Allergy and Immunology, Vaccine Discovery, La Jolla, California, United States of America; Singapore Immunology Network, Singapore

## Abstract

**Background:**

A significant fraction of the more than 18 million scientific articles currently indexed in the PubMed database are related to immune responses to various agents, including infectious microbes, autoantigens, allergens, transplants, cancer antigens and others. The Immune Epitope Database (IEDB) is an online repository that catalogs immune epitope reactivity data derived from articles listed in the National Library of Medicine PubMed database. The IEDB is maintained and continually updated by monitoring PubMed for new, potentially relevant references.

**Methodology:**

Herein we detail the classification of all epitope-specific literature in over 100 different immunological domains representing Infectious Diseases and Microbes, Autoimmunity, Allergy, Transplantation and Cancer. The relative number of references in each category reflects past and present areas of research on immune reactivities. In addition to describing the overall landscape of data distribution, this particular characterization of the epitope reference data also allows for the exploration of possible correlations with global disease morbidity and mortality data.

**Conclusions/Significance:**

While in most cases diseases associated with high morbidity and mortality rates were amongst the most studied, a number of high impact diseases such as dengue, Schistosoma, HSV-2, B. pertussis and Chlamydia trachoma, were found to have very little coverage. The data analyzed in this fashion represents the first estimate of how reported immunological data corresponds to disease-related morbidity and mortality, and confirms significant discrepancies in the overall research foci versus disease burden, thus identifying important gaps to be pursued by future research. These findings may also provide a justification for redirecting a portion of research funds into some of the underfunded, critical disease areas.

## Introduction

More than 18 million scientific articles are currently indexed in the PubMed database.

A significant number of these relate to epitopes associated with immune responses to various agents, including infectious microbes, autoantigens, allergens, transplants, cancer antigens and others. The relative number of references in each category reflects the current and past research focus on immune reactivities. Here we analyze the data from these different domains to evaluate overall epitope data coverage therein, and highlight the strengths and weakness in our overall knowledge base.

The Immune Epitope Database (IEDB) is an online repository of manually curated data, which catalogs immune epitope reactivity data [www.immuneepitope.org]. The PubMed database is constantly queried for potentially relevant references. To capture the highest possible fraction of references describing immune epitope data, these queries are intentionally broad. To date more than 21,000 references of potential interest (>1% of PubMed) have been identified for inclusion into the IEDB. Their potential relevance is determined first by an automated text classifier based on the information contained within their abstract, title and journal title [1], and then manually by subject matter experts. The criteria for passing this initial selection process require that *i*) the reference contains experimental data describing adaptive immune responses, *ii*) the data is original (for example, review papers and use of epitopes as a mere marker or tag are excluded), and *iii*) the epitope molecular structure is described in sufficient detail [2]. Following these preliminary determinations, a systematic review of the paper is performed, and if the study is still deemed to contain curatable information, the data are extracted, entered into the database and the curated record then becomes available to the public.

## Results

### A broad-net strategy to capture epitope-related literature

Currently, four different immunological domains are recognized and prioritized for curation within the IEDB. As first priority, Infectious Diseases and Microbes have been targeted, followed by Allergy, Autoimmunity and Transplantation. Accordingly, the curation of all references from Allergy and Infectious Diseases is nearly complete (>80% and 90%, respectively). In order to enhance the curation prioritization process, we recently further categorized all potential references into six main immunological domains, or classes. These classes include the four original domains: Infectious Disease and Microbes (excluding HIV), Allergy, Autoimmunity and Transplantation, plus HIV (including SIV and related viruses) and Cancer. References containing immune epitope data, but not pertinent to any of the main classes were categorized as “Other”. This detailed classification allows not only for enhanced prioritization, but also enables a more accurate/thorough description and accounting of the IEDB reference data.

**Figure 1:**
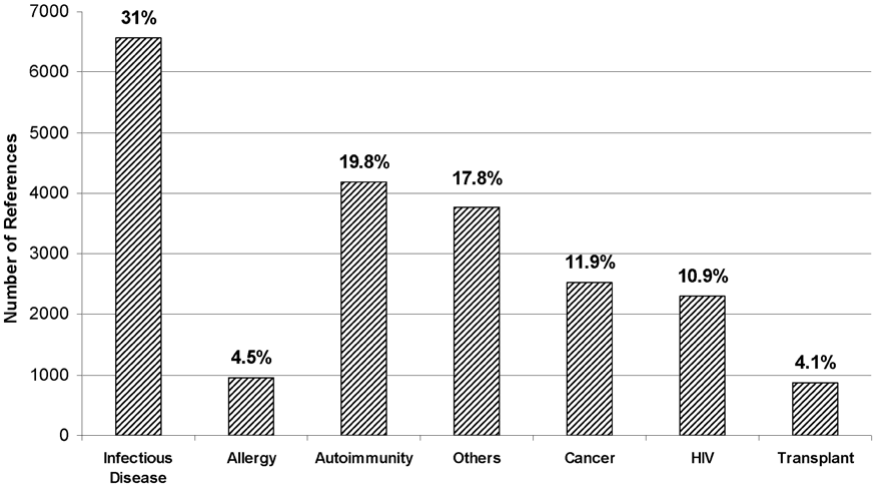
Distribution of references within main classes. The data represent the distribution of the 21,269 total potentially relevant references by percentage (%) and total number within each main class.

The relative number of references classified in each of these main classes is shown in Figure 1. It can be seen that Infectious Diseases and Microbes represent the majority of references, accounting for 31% of the references. Together with HIV references (∼11%), Infectious Diseases therefore represent more than 42% of the immune epitope literature.

Next, Autoimmunity and “Other” references make up nearly 20% each. Cancer references represent nearly 12% of the references, and finally, the least references are available for the Allergies and Transplantation classes (less than 5% each). Each main class was next parsed in a finer set of categories.

### A detailed breakdown of the pathogens associated with Infectious Diseases/and Microbes references

**Figure 2:**
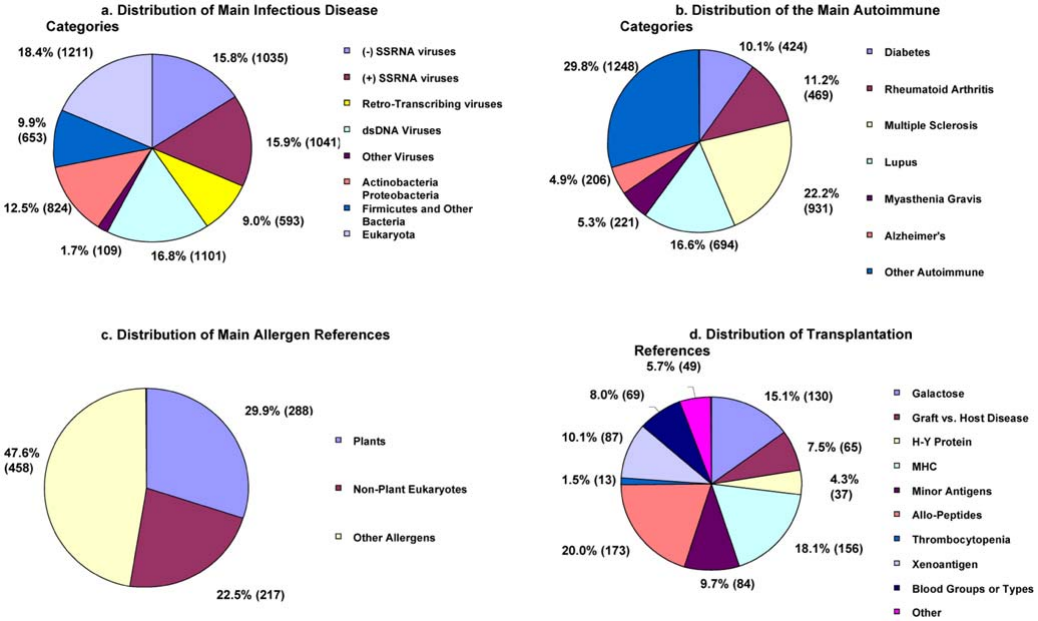
Distribution of Main Infectious Disease Categories. The various pie charts represent the percentage (%) and total number of references (in parentheses) of different categories within each main class. A) Infectious Disease and Microbes (non-HIV) B) Autoimmunity C) Allergy D) Alloantigens and Transplantation.

Using the established NCBI taxonomy, eight major categories were defined within the Infectious Diseases and Microbes class: 1) negative strand RNA viruses, 2) positive strand RNA viruses, 3) retro-transcribing viruses [Retroviridae, Hepadnaviridae], 4) double-stranded DNA (dsDNA) viruses, 5) Other viruses, 6) Actinobacteria and Proteobacteria 7) Other bacteria, and 8) eukaryotic organisms. Each of these categories corresponds to different phylogenetic groupings designed to encompass approximately all related references, with the exception of the “Other viruses” category, which contains approximately 109 references (Figure 2a). Each category was further classified in several subcategories (Table S1).

The overall greatest representation was from viral pathogens (59.2%), followed by bacterial pathogens (22.4%) and parasites (18.4%). Looking at the individual categories, the majority of references within negative-strand RNA viruses, are derived from influenza viruses (52%), followed by viruses within the Paramyxoviridae family (RSV, Measles, Mumps; 25.5%) [Table S1]. Within positive-strand RNA viruses, the hepatitis C virus (HCV) subcategory accounts for about 34.1% of the references, followed by Picornaviruses (poliovirus, coxsackievirus, foot and mouth disease virus; 26.3%). The Flaviviridae (West Nile, yellow fever virus and dengue virus) and Nidovirales (SARS and other coronaviruses) families are less well represented, representing 8.5 and 13.2%, respectively. In the retro-transcribing virus category, excluding Lentiviruses (HIV, SIV) which are separately classified by another group in the HIV Los Alamos database [www.hiv.lanl.gov], the delta retrovirus (HTLV) and hepatitis B virus (HBV) represent the bulk of the category, with 22.1% and 53.5%, respectively. In the category of dsDNA viruses, Herpesviridae are by far the most represented. Specifically, there are 185, 208 and 233 references each for alpha, beta and gamma herpesviruses, respectively, accounting for 57% of the category. Another 315 references are related to human papilloma virus (HPV). Finally, relatively few (45) references are related to poxviruses.

Within the Actinobacteria/Proteobacteria category, references related to Mycobacteria are by far the most numerous (39.6%), followed by Enterobacteria (E.coli, Salmonella, Yersinia, Shigella and Proteus). Fewer references are related to other classes containing important human pathogens, such as bacteria in the genera Vibrio, Haemophilus, Pseudomonas, Anaplasmas, Neisseria and Bordetella. Likewise, in the category of Firmicutes/Other Bacteria, well-represented categories include Listeria, Streptococci and Clostridiales. References describing epitopes from Staphylococcus, Chlamydia, Spirochaetes and other bacteria are less well represented. Finally, in terms of parasitic eukaryotic organisms, the most references by far are represented as relating to Plasmodium [malaria] (48.1%); followed by Eukaryotic invertebrates such as Nematodes, Platyhelminthes, and Schistosomas (18.8%). Fewer references are represented by other eukaryotes in this category, such as Entamoeba, Theliera and Babesia.

### A classification of Autoimmunity references based on a combination of associated disease and autoantigens

Epitope-specific references that were broadly related to autoimmunity were classified in seven categories, based on the specific type of autoimmune manifestations: 1) diabetes 2) rheumatoid arthritis, 3) multiple sclerosis, 4) lupus, 5) myasthenia gravis, 6) beta amyloid reactions/Alzheimer's disease, and 7) other autoimmune diseases. A pie chart representing the relative distribution of these categories is shown in Figure 2b. As it can be seen, multiple sclerosis is by far the most represented class, with 22.2% of the references, followed by the rheumatoid arthritis, diabetes and lupus categories all represented by similar numbers of references in the 10 to 17% range. Each of these categories was further sub-categorized. These sub-categories were mostly organized on the basis of the protein or molecular structure recognized by immune responses [Table S2].

Within the MS references, those relating to myelin oligodendrocyte glycoprotein (MOG), proteolipid protein (PLP) and myelin basic protein (MBP) in aggregate represent 84% of the references, and that references presenting data related to other target antigens only encompass ∼16% of the total. More specifically, three subcategories relate to myelin basic protein (MBP), and these collectively encompass ∼68% of the MS related references. References relating to the most well-characterized epitopes/regions (MBP 78, MBP 1–9 and PLP 139–151) out-numbered references describing all other epitopes derived from the same two MS target antigens. We have chosen to classify these references separately, and only a few representative references might be curated in the IEDB for each epitope; references presenting largely redundant data may be excluded or considered of lower priority.

A different picture emerges in the case of lupus-associated references, where a myriad of different targets are reported. Here, epitopes are derived from antigens of different chemical types (proteins, lipids and DNA). With the exception of antiphospolipid and anti-cardiolipin associated references, which represent ∼44% of the references, no other class of antigens appear to be greatly over-represented in comparison to the others.

Within diabetes references, two subcategories encompass the majority of the references, namely those related to proinsulin/insulin and glutamic acid decarboxilase (GAD), respectively. Similarly, in the case of rheumatoid arthritis, eight different reference subcategories were defined, with most references belonging to collagen. The next most represented subcategories were citrullinated epitopes, heat shock proteins and rheumatoid factors/antibodies.

Finally in the broad class of ‘other’ autoimmune references, nine main subcategories were defined ranging from reactions against reproductive antigens (investigated as potential contraceptive measures), anti-interferon and anti-von Willebrand factor reactions (mostly associated with reactivity resulting from treatment with protein therapeutics) and organ-specific autoimmune manifestations (thyroid, liver, and uveitis). This class constitutes a large percentage of the total autoimmune class, representing some 1248 references.

### The Classification of Allergy references

Allergy references were classified in 3 main categories based on the source of the allergen itself: plants (288 references), animals and fungi (217 references), and other (458 references). A pie chart representing the relative distribution of these categories is shown in Figure 2c. Each of these categories was further classified in subcategories [Table S3].

Within the Plant category, we further distinguish eight main subcategories. Approximately one quarter of these references are related to gluten and celiac disease.

The second most populated subcategories are those related to Betulaceae (the birch family), Cupressaceae (the cypress and cedar family) and Poaceae (mostly timothy grass). Less frequently populated are the subcategories corresponding to latex and to the Fabaceae family (soybean and peas), as well as ‘other trees’ and ‘other flowering plants.’

The animal and fungi category is further broken down in various subcategories, corresponding to allergies to arachnids (mites and ticks) 29.5%, and insects 18.4%, and fungi 12%. A significant number of references are also identified corresponding to allergens derived from vertebrates, such as animal dander or food products (i.e. milk). Here, mammals and birds account for 26.7% and 6%, respectively, of the references in the Eukaryote (Non-plant) category.

Finally, within the ‘other allergens’ category, most references relate to small, non-peptidic experimental allergens involved in hypersensitivity reactions and/or commonly utilized as model small molecule allergens (haptens). Of these, nearly half involve dinitrophenol (DNP) and related molecules, and a much smaller fraction relates to metals (nickel, beryllium and others). Other haptens encompass the remaining 40% or so of the references.

### The classification of Alloantigen and Transplantation references

The main class of Transplantation/allorecognition represents the smallest class and a fairly heterogeneous set of references. A relative large fraction of these references is related to galactose (15.1%), an important determinant recognized by rejection-associated antibodies. Other related categories included xenotranplantation (10.1%) and blood groups (8%). Also very prominent is the category of references related to MHC molecules (18.1%), which represent important targets, either as a whole protein, or as a source of allorecognized peptides. Other allopeptides include the minor histocompatibility antigen (HY protein) category (4.3%), generic allopeptides (20%), and minor antigens (9.7%). Finally, two additional categories represent epitopes associated with specific disease settings such as thrombocytopenia and graft-versus-host disease (graft rejection). A pie chart representing the relative distribution of these categories is shown in Figure 2d and Table S4 provides a breakdown of these categories by number of references.

### The ‘other’ references

To avoid duplication of ongoing efforts at the HIV Los Alamos database [www.hiv.lanl.gov], references related to HIV, SIV and other Lentiviruses are presently not included within the scope of the IEDB. Likewise, cancer references involving specific immune epitopes are not currently considered within NIAID's priority, and accordingly, their categorization is not described herein. The final main class, “Other,” was designed as a catchall for those references not conforming to any of the above organism- or disease-based groupings. However, perusal of the references within this class helps complete the picture of molecular targets related to adaptive immune responses.

These 3,765 references were further sub-dived in eight categories (Table 1). The most numerous categories were related to non-disease-related, non-peptidic antigens, such as the DNP hapten (7.8%), carbohydrate epitopes (2.5%), gangliosides (5.1%), and other small molecular/haptens (24%). References related to the definition of epitopes recognized by monoclonal antibodies (20.8%), and by other non-monoclonal B cell responses (8.8%) are also rather numerous. Additional categories capture model antigens, like cytochrome C (Cyt C), hen egg white lysozyme (HEL) and class II-associated invariant chain peptides (CLIP). A large number of papers also exist in the scientific literature relating to ovalbumin (OVA) epitopes, specifically OVA 257–264 (SIINFEKL) and OVA 323–339 (ISQAVHAAHAEINEAGR). Representative references for these two epitopes have been, or are in the process of being captured. In most other instances these epitopes are utilized as ‘tags’ and thereby are likely to be excluded in the database. Finally, other categories in this class relate to naturally processed ligands eluted from MHC molecules, (5.8%), epitopes defined by X-ray crystallography, NMR structures (3.3%), and definition of MHC binding motifs (4.6%).

**Table 1:** Classification of “Other” References.

Category		# of References	% of Total
**Non-Peptidic Antigens**			
DNP, TNFB, TNP, TNCB		293	7.8%
Other Haptens		905	24.0%
Galactose (Sugars)		94	2.5%
Gangliosides		192	5.1%
	**Total**	1484	39.4%
**Model Antigens**			
Class II-associated Invariant Chain Peptides		28	0.7%
Lysozyme (HEL)		200	5.3%
Myoglobin		58	1.5%
Cytochrome C, Other Cytochromes		128	3.4%
Analog, Antagonist		62	1.6%
	**Total**	476	12.7%
Monoclonal Antibodies		783	20.8%
B Cell Other		331	8.8%
T Cell Other		175	4.6%
Motifs		172	4.6%
Structure		126	3.3%
Naturally Processed		218	5.8%
	**Total**	1805	47.9%
	**Grand Total**	3,765	100.0%

Each subcategory describes the number of references, as well as the percentage with regard to the absolute total number of “Other” references.

### Time course of reference deposition

**Figure 3:**
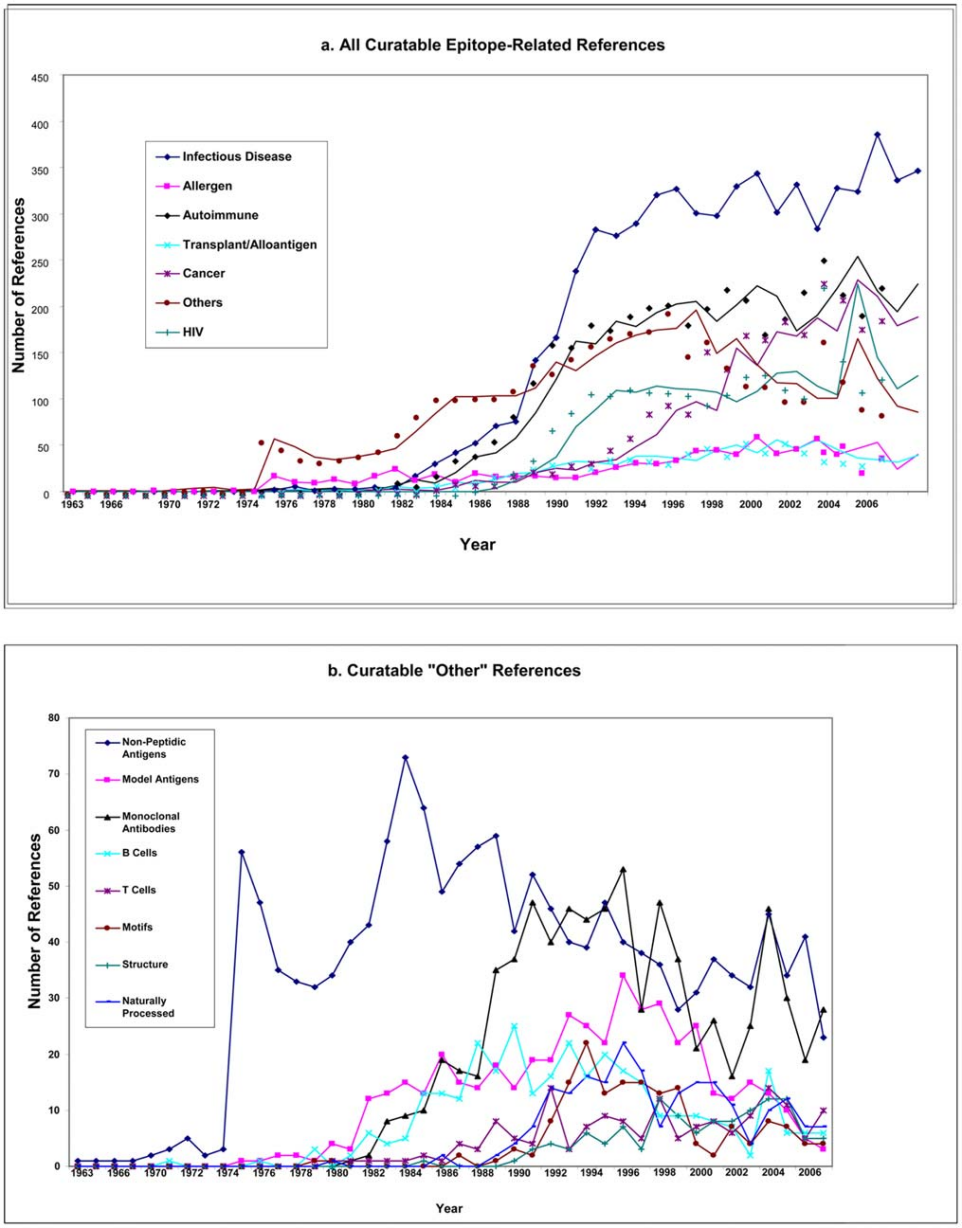
Time course of reference deposition. A) The number of epitope-related publications is plotted against year of publication for each different class. HIV data are shown separately from infectious disease to avoid duplication of ongoing efforts at the HIV Los Alamos database [www.hiv.lanl.gov]. B) The number of epitope-related publications is plotted against year of publication for each different category within the “Other” class.

Having completed the categorization of all references, we investigated the time course of publication. Figure 3a shows the rate of new publication describing epitope data as appearing in the scientific literature between 1960 and the current year. While a negligible number of publications were observed between 1960 to the mid 1970's, a sudden jump in the number of publications occurred thereafter. This increase in epitope-related literature is likely related to the discovery and utilization of monoclonal antibodies. A further remarkable jump in publications occurs in the mid to late 1980s', probably related to the demonstration that small peptides are the ligands recognized by MHC molecules and T cells. Epitope references for HIV increased steadily starting in the late 1980s, reaching a peak in the early 90s that leveled off and remained fairly constant thereafter.

In general, all categories follow a similar trend, with the exception of the ‘other’ class, which appears to start accumulating references sooner than the others, but also appears to level off and even decrease in rate in more recent times. To investigate the reason for this profile, we plotted the rate of publication of each of the subcategory, as shown in Figure 3b. This analysis revealed a rather complex picture. It was found that references in PubMed relating to non-peptidic antigens were responsible for the initial jump during the mid-1970s, but declined steadily after peaking in the mid 1980s. References relating to model protein antigens start to appear in the early 80s, peak in 1999 and decline sharply thereafter. Likewise, references related to definition of epitopes recognized by polyclonal antibodies peak in the early 1990s and then decline, in concomitance with the rise, also in the 90s, of references describing epitopes recognized by monoclonal antibodies. Likewise, references relating to definition of MHC motifs and elution of naturally processed ligands, peak in the mid 90s and decline thereafter.

### Correlation with societal impact

The categorization of immune epitope data presented herein relates to domains such as Infectious Diseases and Microbes, Autoimmunity, Allergy and Transplantation. It may therefore be taken as representative of the degree to which the molecular targets associated with these diseases have been scrutinized and defined. An immune epitope is defined by the IEDB as the molecular structure interacting with receptors of the immune system (T cell and B cell/antibody). Accordingly this definition excludes, for example, structures involved in lectin-carbohydrate interactions, or structures recognized by receptors involved in innate immunity, such as NK cells. Immune epitopes are defined in the literature with varying level of resolution. In some cases the minimal/optimal residues of a T or B cell determinant are exactly defined, while in other cases only certain key components are mapped. Finally, in certain instances broad regions are pinpointed as containing the epitope, but the exact boundaries and components are not defined. No structures >50aa are considered for inclusion in the IEDB. Because it is difficult to adequately compare the societal impact (i.e. disease burden in morbidity and mortality) of vastly different diseases categories such as Allergies, Cancer, Autoimmunity or Infectious Diseases, we have elected to investigate the overall impact of disease burden within only the infectious and autoimmune disease classes. For this purpose, we examined global morbidity (prevalence) and mortality data for a representative list of pathogen/diseases, and then compared these data to the number of references in each category.

A total of thirty infectious diseases were selected based on the most prevalent disease categories for which sound epidemiological data existed. Table 2 shows the ranking of each pathogen/disease according to the total number of immune epitope references and the estimated morbidity (prevalence) and mortality for each. The ten pathogens associated with the most references are highlighted in gray; the numbers in bold highlight the top ten pathogens/diseases with respect to prevalence and mortality. The ranking of disease by prevalence or mortality is also indicated in parentheses (1–10). The top ten diseases by overall reference abundance are HIV/AIDS, influenza, Malaria, HCV, HBV, HPV, TB, group A Streptococcus, Measles and RSV.

**Table 2:** Ranking of Epitope References by Infectious Disease Burden.

Rank	Pathogen/Disease	Refs	Cases	Deaths
1	HIV/AIDS	2,297	33,000,000	**^(1)^ 2,000,000**
2	Influenza	548	**^(1)^ 600,000,000**	**^(8)^ 375,000**
3	Malaria	483	**^(5)^ 247,000,000**	**^(4)^ 881,000**
4	Hepatitis C	356	**^(6)^ 170,000,000**	54,000
5	Hepatitis B	323	**^(3)^ 350,000,000**	**^(3)^ 900,000**
6	HPV (Cervical Cancer)	282	500,000	**^(9)^ 240,000**
7	TB (Active)	264	14,400,000	**^(2)^ 1,700,000**
8	GAS	178	18,000,000	**^(5)^ 500,000**
9	Measles	93	279,000	197,000
10	RSV	92	**^(9)^ 64,000,000**	160,000
11	Typhoid fever (S. typhi)	86	19,000,000	**^(10)^ 216,000**
12	Schistosoma	74	**^(4)^ 261,000,000**	41,000
13	Dengue	68	**^(8)^ 100,000,000**	22,000
14	Polio	59	1,600	NA
15	T. cruzi	58	6,500,000	52,000
16	C. trachomatis	57	**^(7)^ 140,000,000**	9,000
17	N. meningitidis	55	1,700,000	170,000
18	Leprosy	54	212,000	5,400
19	Leishmania	51	12,000,000	47,000
20	Haemophilus (HiB)	44	3,000,000	**^(7)^ 386,000**
21	Rabies	44	10,000,000	55,000
22	HSV-2	38	**^(2)^ 536,000,000**	NA
23	V. cholerae	33	3,000,000	120,000
24	Rubella	31	836,000	20,000
25	West Nile	21	29,000	1,070
26	B. pertussis	16	**^(10)^ 60,000,000**	**^(6)^ 400,000**
27	Yellow Fever	16	200,000	30,000
28	N. gonorrhoeae	15	52,000,000	1,000
29	Ebola	13	2,000	1,200
30	Mumps	7	3,000,000	300

Diseases are ranked (1–30) according to the number of immune epitope references (related to infectious disease) identified in PubMed. The number in parentheses indicates their respective ranking (1–10).

In general, the majority of pathogens listed in the top ten most prevalent in mortality have more than 100 references each, and represents diseases responsible for morbidity in hundreds of millions of people per year. While it is perhaps not surprising that the epitope reference coverage would be high for prominent infectious diseases [3], the length of the list of diseases for which coverage was poor (<80 references) is surprising, as it includes five very high impact diseases (dengue, Schistosoma, HSV-2, *B. pertussis* and *Chlamydia trachoma*). Indeed, *B. pertussis* stands out prominently, with some 60 million people infected per year and an estimated 400,000 deaths, and a mere 16 references.

**Figure 4:**
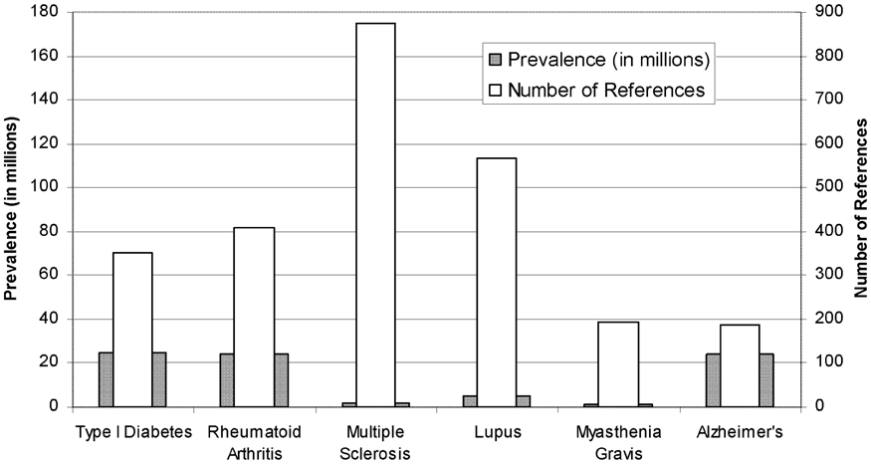
Morbidity Data for Autoimmune Diseases. Prevalence data for Type I diabetes is an extrapolation from total diabetes cases. Type I diabetes represents only 10% of the total, which was reported as being 180,000,000 by the WHO.

Figure 4 shows the coverage for autoimmune diseases described by the immune epitope data. Interestingly, it seems that type I diabetes and RA, despite being associated with higher morbidity and/or mortality as compared to MS and lupus, are actually associated with relatively lower numbers of references. It would thus appear that a relative imbalance of epitope knowledge might also exist in the Autoimmunity class, when analyzed in the context of disease prevalence.

In the past [4], we have also investigated overall epitope coverage at the genomic level by calculating the total number of proteins from which epitopes have been derived and the percent of the genome by antigen targeted for epitope identification. Applying this approach to the current analysis, we calculated coverage for high relevance/low reference coverage pathogens and compare these to high profile pathogens (Table 3). In the majority of cases, pathogens well-represented in the literature have greater epitope coverage, whereas those less well-represented have lower epitope coverage. While the epitope coverage was greater for high profile pathogens like TB and Malaria, the actual percentage itself was rather low (ex. 7% for TB and 0.9% for Malaria). Furthermore, high overall coverage does not necessarily translate in well-balanced coverage. In many cases, the majority of the identified epitopes come from very few antigens. This phenomenon was observed and previously discussed in detail for high profile pathogens such as *P. falciparum*, TB and influenza [4]–[6]. These results further emphasize how the current epitope literature is far from complete and how numerous gaps and opportunities for further investigation still exist.

**Table 3:** Epitope Coverage.

Pathogen	Coverage in Literature	Number of Proteins with Defined Epitopes	Total Number of ORFs/Expressed Proteins	Percent (%)
TB	HIGH	270	3,900	7
B. pertussis	LOW	6	3,800	0.2
Malaria	HIGH	46	5,000	0.9
S. mansoni	LOW	15	12,880	0.1

Overall epitope coverage was assessed by comparing the coverage in the literature (total number of references), which was defined as high or low, and the precentage of the genome (or total ORFs) represented by reported epitopes to date.

## Discussion

The data presented herein for the first time allows for a glimpse of the balance of references contained in the scientific literature, related to epitope data and to all associated immunological domains, including Infectious Diseases and Microbes, Autoimmunity, Allergy, Transplantation and Cancer. Several conclusions emerged from the analysis of the data gathered so far.

First, it is of interest to note the relative distribution of the references among the domains, or classes. By far the majority of references are related to Infectious Diseases and Autoimmunity. Cancer is a distant third, and relatively fewer references are available for Transplantation and allergies. It is unclear at this stage whether this reflects a differential focus of the scientific community, or inherent difficulties in defining the molecular targets (epitopes) recognized by adaptive immune responses in those settings. Interestingly, within the Infectious Disease class, we found that references representing viral pathogens outnumber bacterial and parasitic pathogens by about 3 to 1. This is likely due to biological factors relating to genome size and/or antigenic complexity. The autoimmune class is dominated by MS references and, not surprisingly, the Allergy class shows a majority of references defining plant allergens. References related to Transplantation are mostly focused on allo-antigens/major histocompatibility complexes.

In addition to describing the overall distribution of data within the six classes, this characterization of the epitope reference data has also allowed us to explore possible correlations with global morbidity and mortality. Here, we investigated the relationship between epitope coverage and overall disease burden by comparing the top ten pathogens/diseases in terms of the total number of references with the total number of cases and deaths per year worldwide. This analysis showed that while most of the high impact infectious diseases (in terms of prevalence and deaths) were well-represented in the top ten, several very significant diseases were represented by far fewer references, thus highlighting a significant imbalance in the coverage. Examples of these high relevance/low coverage infectious diseases are dengue, Schistosoma, HSV-2, *B. pertussis* and *Chlamydia trachoma*. Similar observations were made for diseases within the autoimmune domain, where the relatively lower impact diseases MS and lupus have much greater epitope coverage than type 1 diabetes and RA. These gaps are even more significant in light of our recent findings following in-depth meta-analyses of some of the most well studied diseases, such as influenza, TB and malaria [4]–[7], which revealed significant gaps and imbalances within the immunological knowledge associated with each of these pathogens.

A sub-analysis of several representative infectious diseases undertaken to examine epitope coverage at the genomic level showed similar results. In the majority of cases, pathogens well-represented in the literature had greater epitope coverage, as measured by the number of ORFs targeted for epitope identification, whereas those less well-represented had lower epitope coverage. However, while the epitope coverage was greater for high profile pathogens like TB and Malaria, the actual percentage itself was rather low. This overall lack of coverage for these pathogens is likely due to a combination of factors, including genome size and organism complexity. Indeed, the genome of *M. tuberculosis* is ∼4,000 ORFs, and the etiological agent of malaria, *P. falciparum*, has an even larger genome and a complex 3-stage life cycle. In addition, we found that good coverage did not necessarily translate in well-balanced coverage, as in many instances, the bulk of epitope identification focuses on just a few of the total antigens. This observation has been made previously by our group, the best example of which is malaria. Here we found that the vast majority of epitope identification was focused on the circumsporozoite surface protein (CSP), despite the 5,000+ ORFs in existence [6].

These results have relevance to the ongoing debate as to whether the direction of biomedical research accurately reflects the reality of the global disease burden [8]. A recent report [9] analyzing the global burden of major infectious diseases indicated that R&D funders have focused overwhelmingly on TB, Malaria and HIV/AIDS, while other diseases with an even bigger burden get little R&D attention. Indeed, numerous studies have already highlighted this trend [10], [11]. According to another report from an international research foundation [12], less than 10% of the world's research budget is spent on conditions that account for 90% of global disease. Our data represents the first estimate of how knowledge in terms of immunological data, rather than R&D funding corresponds to disease morbidity and mortality, and confirms significant discrepancies in the overall research focus versus disease burden, thus identifying important gaps to be pursued by future research.

One important issue that became apparent upon embarking in the classification of the overall “universe” of epitope-specific references is that not all classification schema are applicable to the various broad domains of immunological literature. In the case of infectious diseases, we utilized the NCBI taxonomy as a guide in the categorization.

However, in the case of autoimmunity, the classification is not related to the organism from which the epitope is derived, but rather from a broad classification of the associated autoimmune disease manifestation. Furthermore, within each autoimmune category, sub-categories were mostly organized on the basis of the protein or molecule recognized.

This approach was taken to reflect the classification utilized by most scientists operating in this field. The classification of allergy references also required development of a similar approach, because a classification based on the organism source of the allergen would not conform to the established classification in use in the allergy community. Here we were able to rely on the backbone of the newly formalized immunological ontology to help establish meaningful categories and sub-categories.

Finally, we would like to point out how the current data is by necessity preliminary, as the accuracy of the classification of the references is dependent on the stage of the curation process. For example, in our experience about 30% of the references deemed potentially acceptable on the basis of the abstract, are eventually disqualified from curation because they fail to meet all inclusion criteria. Thus approximately 70% of targeted references become incorporated into the database. We would also like to underscore that the work presented herein is tightly linked to other efforts currently ongoing with the IEDB. Specifically, the present work benefits from the development of formal ontologies and classification of immune epitope data [13], and at the same time informs further development of ontologies and controlled vocabularies, as new reference categories are curated. Most importantly, the current work has crucially relied on the development of automated text classifiers [1], and in turn the present data can be utilized to further develop new methods of automated text classification and characterization.

## Materials and Methods

Worldwide morbidity and mortality figures were obtained, in large part, from the WHO using their publically available data. The WHO draws on a wide range of data sources to quantify global and regional effects of diseases, the details of which are not discussed here, however are available on their websites [Tables S5 and S6] (www.who.int). When statistics were not available through the WHO, we used the peer-reviewed literature and/or personal communication with experts in the respective fields. Further validation was sought for these figures by contacting subject matter experts (SMEs) to confirm the accuracy of estimates and timeframes. Because morbidity and mortality data for individual pathogens/diseases are acquired at different yearly intervals, data derived from a single year could not be determined for all of the pathogens included herein. The majority of the figures were available for years 2004–2008, however, some figures were older than 2004 and have been noted. Accurate and reliable burden of disease estimates are currently difficult to assess as a result of inconsistent data collection and/or reporting, especially in the developing world. Therefore, the majority of the data shown presented herein are likely underestimates. The majority of the prevalence data represents case estimates by the WHO, unless otherwise noted as actual reported cases.

To accomplish this sub-analysis, we first assembled a consolidated list of 30 pathogens to target from all the infectious disease categories described in Table 1. These were selected based on the most prevalent disease categories for which sound epidemiological data existed. For example, within the broader categories such as single stranded, positive sense RNA viruses, we selected prominent pathogens such as measles virus, RSV, Mumps, rabies virus, Ebola virus and all influenza viruses as representative of the group. Preliminary prevalence and mortality figures were then assigned to each pathogen using the WHO web page (WHO fact sheets). In order to verify that our data corresponds with what is currently accepted within the scientific community a list of subject matter experts (SME) was generated using the ISI web of knowledge site [apps.isiknowledge.com]. Under the Web of Science tab, the terms “morbidity or prevalence or mortality and disease x” were applied in order to flag the top 5 experts within each disease-specific field based on their total number of publications. Subject matter experts were then formally contacted by email to seek independent validation of the preliminary figures. Prevalence and mortality numbers were then adjusted, if warranted, to those provided by the SMEs. On average, three authors were emailed for each disease in order to obtain validation. Diseases, for which reliable figures could not be found and/or were not available through the SMEs, were excluded from this part of the analysis.

## Supporting Information

Table S1 This represents a summary of each major infectious disease category, excluding HIV, showing subtype designations to the specificity of the source organism's genus. There are 6,567 infectious disease references, which are distributed across the main infectious disease categories. The percentage column indicates each category as a percent of the total amount of infectious disease references.
(0.09 MB DOC)

Table S2 This represents a summary of the various autoimmune main categories, as well as their specific subcategory designations. These subcategories were organized on the basis of the actual protein or molecular structure recognized by immune responses. The percentage column indicates each category as a percent of the total amount of autoimmune references.
(0.08 MB DOC)

Table S3 There are three main allergy categories and these were further classified into subcategories. The main plants category contains trees, plants and grasses. The non-plant eukaryotes contain insects, mammals, birds, invertebrates and fungi. The other allergens mainly consist of low molecular weight, non-peptidic chemicals and haptens, as well as metals. The percentage column indicates each category as a percent of the total amount of allergy references.
(0.05 MB DOC)

Table S4 Table S4 describes the breakdown of the various alloantigen/transplant subcategories. There are a total of 701 transplant-related journal publications, constituting the lowest represented class. Each subcategory is presented as a percentage of the total.
(0.03 MB DOC)

Table S5 Worldwide morbidity and mortality figures were obtained, in large part, from the WHO using their publically available data. The WHO draws on a wide range of data sources to quantify global and regional effects of diseases, the details of which are not discussed here, however are available on their websites. When statistics were not available through the WHO, we used the peer-reviewed literature and/or personal communication with experts in the respective fields. Morbidity and mortality data for individual pathogens/diseases are acquired at different yearly intervals, data derived from a single year could not be determined for all of the pathogens included herein. The majority of the figures were available for years 2004–2008, however, some figures were older than 2004 and have been noted.
(0.06 MB DOC)

Table S6 Worldwide morbidity and mortality figures were obtained, in large part, from the WHO using their publically available data. The WHO draws on a wide range of data sources to quantify global and regional effects of diseases, the details of which are not discussed here, however are available on their websites. When statistics were not available through the WHO, we used the peer-reviewed literature and/or personal communication with experts in the respective fields. Morbidity and mortality data for individual pathogens/diseases are acquired at different yearly intervals, data derived from a single year could not be determined for all of the pathogens included herein. The majority of the figures were available for years 2004–2008, however, some figures were older than 2004 and have been noted.
(0.04 MB DOC)

## References

[1] Wang P, Morgan AA, Zhang Q, Sette A, Peters B (2007). Automating document classification for the Immune Epitope
Database.. BMC Bioinformatics.

[2] Vita R, Vaughan K, Zarebski L, Salimi N, Fleri W (2006). High-throughput curation of complex, context-dependent
immunological data.. BMC Bioinformatics.

[3] WHO: www.who.int/mediacentre/factsheets/fs310/en/index.html. ‘Top Ten causes of death, 2004.’

[4] Blythe M, Zhang Q, Vaughan K, de Castro R, Salimi N (2007). An analysis of the epitope knowledge related to Mycobacteria.. Immunome Res.

[5] Bui HH, Peters B, Assarsson E, Mbawuike I, Sette A (2007). Ab and T cell epitopes of influenza A virus, knowledge and
opportunities.. Proc Natl Acad Sci U S A.

[6] Vaughan K, Blythe M, Greenbaum J, Zhang Q, Peters B (2009). Meta-Analysis of immune epitope data for all Plasmodia: overview
and applications for malarial immunobiology and vaccine-related issues.. Parasite Immunol.

[7] Assarsson E, Bui HH, Sidney J, Zhang Q, Glenn J (2008). Immunomic analysis of the repertoire of T-cell specificities for
influenza A virus in humans.. J Virol.

[8] Flory JH, Kitcher P (2004). Global health and the scientific research agenda.. Philos Public Aff.

[9] Enserink M (2009). Some neglected disease are more neglected than others.. Science.

[10] McKenna MT, Zohrabian A (2009). U.S. Burden of disease – Past, present and future.. Ann Epidemiol.

[11] Stuckler D, King L, Robinson H, McKee M (2008). WHO's budgetary allocations and burden of disease.. Lancet.

[12] Research does not reflect global disease burden (2000). Western J Med.

[13] Sathiamurthy M, Peters B, Bui H-H, Sidney J, Mokili J (2005). An ontology for immune epitopes: application to the design of a
broad scope database of immune reactivities.. Immunome Res.

