# Supplementary material for: Classification of the Universe of Immune Epitope Literature: Representation and Knowledge Gaps
Source: PLoS One. 2009 Sep 14;4(9):e6948. doi: 10.1371/journal.pone.0006948 (PMC2747625; doi:10.1371/journal.pone.0006948)
Supplement: Table S1 — This represents a summary of each major infectious disease category, excluding HIV, showing subtype designations to the specificity of the source organism's genus. There are 6,567 infectious disease references, which are distributed across the main infectious disease categories. The percentage column indicates each category as a percent of the total amount of infectious disease references. (0.09 MB DOC) [file pone.0006948.s001.doc]

| **Table S1. Infectious Disease References by Subtype Designation** |  |  |
| --- | --- | --- |
| **Category** | **# of References** | **% Total** |
| **(-) Single Strand RNA Viruses** |  |  |
| Paramyxoviridae (RSV, Measles, Mumps) | 264 | 25.5% |
| Rhabdoviridae (VSV, Rabies) | 78 | 7.5% |
| Other Mononegavirales (Ebola, Borna) | 25 | 2.4% |
| H3N2 Influenza A Subtype | 152 | 14.7% |
| H1N1 Influenza A Subtype | 145 | 14.0% |
| Other Influenza A Subtypes (Not H3N2, H1N1) | 225 | 21.70% |
| Other Orthomyxovirdae (Influenza B/C, Unidentified) | 17 | 1.6% |
| Other ssRNA (-) Stand Viruses (Rift Valley Fever Virus, Hanta, Arenavirus) | 129 | 12.5% |
| Total | 1035 | 100.0% |
| **(+) Single Strand RNA Viruses** |  |  |
| Hepatitis C Virus | 355 | 34.1% |
| Dengue Virus | 69 | 6.6% |
| Flaviviridae (West Nile, Yellow Fever) | 89 | 8.5% |
| Nidovirales (SARS, Coronaviruses) | 137 | 13.2% |
| Picornaviruses (Foot and Mouth, Coxsackie, Poliovirus) | 274 | 26.3% |
| Other ssRNA (+) Strand viruses (Rubella, Hepatitis E, Semliki Forest Virus) | 117 | 11.2% |
| Total | 1041 | 100.0% |
| **Retro-Transcribing Viruses** |  |  |
| Non-HIV Lentivivirus (EIAV, Caprine Lentiviruses) | 73 | 12.3% |
| Deltaretrovirus (HTLV) | 131 | 22.1% |
| Hepatitis B Virus | 317 | 53.5% |
| Other Retroviruses (Mouse Mammary Tumor Virus, Leukemia Viruses, Sarcoma Viruses) | 72 | 12.1% |
| Total | 593 | 100.0% |
| **Double Strand DNA Viruses** |  |  |
| Alphaherpesvirinae (Human Herpesvirus 1/2, Varicellovirus) | 185 | 16.8% |
| Betaherpesvirinae (CMV, Roseolovirus, Muromegalovirus, Human Herpesvirus 5) | 208 | 18.9% |
| Gammaherpesvirinae (Epstein-Barr Virus, Rhadinovirus, Human Herpesvirus 4) | 233 | 21.2% |
| Papillomaviridae (HPV) | 315 | 28.6% |
| Adenoviruses | 25 | 2.3% |
| Polyomaviridae (Simian Vacuolating Virus) | 51 | 4.6% |
| Poxviridae (Vaccinia, Pox) | 45 | 4.1% |
| Other dsDNA Viruses (Caudovirales, African Swine Fever Virus, Baculoviruses) | 39 | 3.5% |
| Total | 1101 | 100.0% |
|  |  |  |
| **Other Viruses** | 109 | 100.0% |
| Total | 109 | 100.0% |
| **Actinobacteria/Proteobacteria** |  |  |
| Mycobacterium (Tuberculosis, Leprosy) | 326 | 39.6% |
| Enterobacteriaceae (E. coli, Salmonella, Yersinia, Shigella, Proteus) | 221 | 26.8% |
| Vibrio (V. cholerae, Other Vibrios) | 36 | 4.4% |
| Other Gammaproteobacteria (Haemophilus, Influenza, Pseudomonas aeruginosa) | 108 | 13.1% |
| Alphaproteobacteria (Rhizobiales, Ricksettias, Anaplasmas) | 33 | 4.0% |
| Betaproteobacteria (Neisseria, Bordetella) | 100 | 12.1% |
| Total | 824 | 100.0% |
| **Firmicutes and Other Bacteria** |  |  |
| Staphylococcus | 36 | 5.5% |
| Listeria | 71 | 10.9% |
| Streptococcus | 177 | 27.1% |
| Other Bacilli (Anthracis, Cereus, Geobacillus, Enterococcus) | 38 | 5.8% |
| Clostridiales (Botulinum) | 98 | 15.0% |
| Spirochaetes (Borrelia, Treponema, Leptospiracaeae) | 66 | 10.1% |
| Chlamydiales (Chlamydia) | 79 | 12.1% |
| Other Bacteria (Boronia, Porphyromonas, Mycoplasmas, Campylobacter) | 88 | 13.5% |
| Total | 653 | 100.0% |
| **Eukaryotes** |  |  |
| Worms (Eukaryotic Invertebrates, Nematoda, Platyhelminthes, Shistosoma, Parasites) | 228 | 18.8% |
| Fungi | 59 | 4.9% |
| Plasmodium (P. falciparum, P. vinckeia, P. yoelli) | 583 | 48.1% |
| Trypanosomatidae (Trypanosoma, Leishmania) | 111 | 9.2% |
| Other Eukaryotes (Entamoebidae, Babesia, Parabasalidea, Coccidia, Theileria, Toxins, Plants) | 230 | 19.0% |
| Total | 1211 | 100.0% |
|  |  |  |
| **Grand Total** | 6567 |  |

Table S1: This represents a summary of each major infectious disease category, excluding HIV, showing subtype designations to the specificity of the source organism’s genus. There are 6,567 infectious disease references, which are distributed across the main infectious disease categories. The percentage column indicates each category as a percent of the total amount of infectious disease references.
