# Supplementary material for: Classification of the Universe of Immune Epitope Literature: Representation and Knowledge Gaps
Source: PLoS One. 2009 Sep 14;4(9):e6948. doi: 10.1371/journal.pone.0006948 (PMC2747625; doi:10.1371/journal.pone.0006948)
Supplement: Table S2 — This represents a summary of the various autoimmune main categories, as well as their specific subcategory designations. These subcategories were organized on the basis of the actual protein or molecular structure recognized by immune responses. The percentage column indicates each category as a percent of the total amount of autoimmune references. (0.08 MB DOC) [file pone.0006948.s002.doc]

| **Table S2. Classification of Autoimmune References** |  |  |
| --- | --- | --- |
| **Category** | **Number of References** | **% of Total** |
| **Diabetes** |  |  |
| Glutamic Acid Decarboxylase | 141 | 33.3% |
| Heat Shock Proteins | 19 | 4.5% |
| Insulin/Proinsulin | 119 | 28.1% |
| Insulinoma-Associated Protein-2 | 26 | 6.1% |
| Islet-Specific Glucose-6-Phosphatase Catalytic Subunit-Related Protein | 15 | 3.5% |
| Various/Multiple Subtypes | 27 | 6.4% |
| Other (Undefined Subtype) | 77 | 18.2% |
| **Total** | **424** | **100.0%** |
| **Rheumatoid Arthritis** |  |  |
| Citrullinated Auto-antibodies | 84 | 17.9% |
| Collagen | 118 | 25.2% |
| Collagen, Type II (260-267; 250-270) | 52 | 11.1% |
| Heat Shock Proteins | 50 | 10.7% |
| Human Cartilage | 20 | 4.3% |
| RA Motif/Shared Epitope | 15 | 3.2% |
| Rheumatoid Factors | 44 | 9.4% |
| Various/Multiple/Other Subtypes | 86 | 18.3% |
| **Total** | **469** | **100.0%** |
| **Multiple Sclerosis** |  |  |
| Anti-Myelin Oligodendrocyte Glycoprotein | 53 | 5.7% |
| MOG (37-48; 35-55; 42-53) | 70 | 7.5% |
| General Myelin Basic Proteins | 299 | 32.1% |
| MBP (78-99; 83-99; 85-99; 83-92; 87-99) | 149 | 16.0% |
| MBP Ac 1-9; 1-10 | 58 | 6.2% |
| Proteolipid Protein (PLP) | 73 | 7.8% |
| PLP 139-151 | 81 | 8.73% |
| T-cell Receptor Peptides | 40 | 4.3% |
| Various/Multiple Subtypes | 38 | 4.1% |
| Other (Undefined Subtype) | 70 | 7.5% |
| **Total** | **931** | **100.0%** |
| **Lupus** |  |  |
| Antibodies | 24 | 3.5% |
| Antiphospholipid/Cardiolipin | 305 | 43.9% |
| DNA, Nucleic Acids | 82 | 11.8% |
| Histones (H1, H3, H4, H2A, H2B) | 39 | 5.6% |
| Inositol 1,4,5-Triphosphate Receptors/NO/Sulfatide antigens | 11 | 1.6% |
| La/SSB | 29 | 4.2% |
| Ribosomal P Proteins (Rib-P) | 16 | 2.3% |
| Ro/SSA | 34 | 4.9% |
| Sm Autoantigen/Small RNP/RNPA | 78 | 11.2% |
| Various/Multiple/Other Subtypes | 76 | 11.0% |
| **Total** | **694** | **100.0%** |
|  |  |  |
| **Myasthenia Gravis** | **221** | **100.0%** |
|  |  |  |
| **Beta Amyloid/Alzheimer's** | **206** | **100.0%** |
|  |  |  |
| **Other Autoimmune Diseases** |  |  |
| Eppin/Luteinizing Hormone/Human Chorionic Gonadotropin/Pellucida | 114 | 9.1% |
| Experimental Autoimmune Myocarditis | 55 | 4.4% |
| Human Interphotoreceptor Retinoid-Binding Protein/Uveitis | 124 | 9.9% |
| Interferons | 46 | 3.7% |
| Liver | 90 | 7.2% |
| Pemphigus/Desmoglein | 51 | 4.1% |
| Thyroid | 180 | 14.4% |
| Von Willebrand/Haemophilia | 121 | 9.7% |
| Other (Undefined Subtype) | 467 | 37.4% |
| **Total** | **1248** | **100.0%** |
|  |  |  |
| **Grand Total** | **4193** |  |

Table S2: This represents a summary of the various autoimmune main categories, as well as their specific subcategory designations. These subcategories were organized on the basis of the actual protein or molecular structure recognized by immune responses. The percentage column indicates each category as a percent of the total amount of autoimmune references.
