# Supplementary material for: Classification of the Universe of Immune Epitope Literature: Representation and Knowledge Gaps
Source: PLoS One. 2009 Sep 14;4(9):e6948. doi: 10.1371/journal.pone.0006948 (PMC2747625; doi:10.1371/journal.pone.0006948)
Supplement: Table S3 — There are three main allergy categories and these were further classified into subcategories. The main plants category contains trees, plants and grasses. The non-plant eukaryotes contain insects, mammals, birds, invertebrates and fungi. The other allergens mainly consist of low molecular weight, non-peptidic chemicals and haptens, as well as metals. The percentage column indicates each category as a percent of the total amount of allergy references. (0.05 MB DOC) [file pone.0006948.s003.doc]

| **Table S3. Classification of Allergy References** |  |  |
| --- | --- | --- |
| **Category** | **Number of References** | **% of Total** |
| **Plants** |  |  |
| Beulaceae (Birch Family) | 40 | 13.9% |
| Cupressaceae (Cypress, Cedar Family) | 30 | 10.4% |
| Other Trees | 19 | 6.6% |
| Fabaceae (Peas, Soybean, Peanut Family) | 22 | 7.6% |
| Latex (Hevea) | 20 | 6.9% |
| Other Flowering Plants | 36 | 12.5% |
| Timothy Grass, Phl, Poaceae | 38 | 13.2% |
| Gluten, Coeliac Disease | 83 | 28.8% |
| **Total** | 288 | 100.0% |
| **Eukaryota (Non-Plants)** |  |  |
| Insecta | 40 | 18.4% |
| Arachnida (Acari, Mites and Ticks) | 64 | 29.5% |
| Mammals | 58 | 26.7% |
| Birds (Aves) | 13 | 6.0% |
| Other Bilateria (Roundworms, Nematodes, Schistosoma, Parasites, Crustacea) | 16 | 7.4% |
| Fungi | 26 | 12.0% |
| **Total** | 217 | 100.0% |
| **Other Allergens** |  |  |
| DNP, DNFB, TNP, TNCB | 183 | 40.0% |
| Other Haptens | 174 | 38.0% |
| Metals (nickel, berillium and others) | 35 | 7.6% |
| Other Allergens | 66 | 14.4% |
| **Total** | 458 | 100.0% |
|  |  |  |
| **Grand Total** | 963 |  |

Table S3: There are three main allergy categories and these were further classified into subcategories. The main plants category contains trees, plants and grasses. The non-plant eukaryotes contain insects, mammals, birds, invertebrates and fungi. The other allergens mainly consist of low molecular weight, non-peptidic chemicals and haptens, as well as metals. The percentage column indicates each category as a percent of the total amount of allergy references.
