# Supplementary material for: Classification of the Universe of Immune Epitope Literature: Representation and Knowledge Gaps
Source: PLoS One. 2009 Sep 14;4(9):e6948. doi: 10.1371/journal.pone.0006948 (PMC2747625; doi:10.1371/journal.pone.0006948)
Supplement: Table S4 — describes the breakdown of the various alloantigen/transplant subcategories. There are a total of 701 transplant-related journal publications, constituting the lowest represented class. Each subcategory is presented as a percentage of the total. (0.03 MB DOC) [file pone.0006948.s004.doc]

| **Table S4. Classification of Alloantigens and Transplantation References** | | |
| --- | --- | --- |
| **Category** | **Number of References** | **% of Total** |
| Galactose | 130 | 15.1% |
| Graft vs. Host Disease | 65 | 7.5% |
| H-Y Protein | 37 | 4.3% |
| Major Histocompatibility Complex | 156 | 18.1% |
| Minor Antigens | 84 | 9.7% |
| Allo-Peptides | 173 | 20.0% |
| Thrombocytopenia | 13 | 1.5% |
| Xenoantigen | 87 | 10.1% |
| Blood Groups or Types | 69 | 8.0% |
| Other | 49 | 5.7% |
| **Total** | **863** | **100.0%** |

Table S4: Table S4 describes the breakdown of the various alloantigen/transplant subcategories. There are a total of 701 transplant-related journal publications, constituting the lowest represented class. Each subcategory is presented as a percentage of the total.
