# Supplementary material for: Classification of the Universe of Immune Epitope Literature: Representation and Knowledge Gaps
Source: PLoS One. 2009 Sep 14;4(9):e6948. doi: 10.1371/journal.pone.0006948 (PMC2747625; doi:10.1371/journal.pone.0006948)
Supplement: Table S5 — Worldwide morbidity and mortality figures were obtained, in large part, from the WHO using their publically available data. The WHO draws on a wide range of data sources to quantify global and regional effects of diseases, the details of which are not discussed here, however are available on their websites. When statistics were not available through the WHO, we used the peer-reviewed literature and/or personal communication with experts in the respective fields. Morbidity and mortality data for individual pathogens/diseases are acquired at different yearly intervals, data derived from a single year could not be determined for all of the pathogens included herein. The majority of the figures were available for years 2004–2008, however, some figures were older than 2004 and have been noted. (0.06 MB DOC) [file pone.0006948.s005.doc]

| **Table S5. Infectious Disease Morbidity and Mortality Sources** | | | | |
| --- | --- | --- | --- | --- |
| **(**-**) sense, SS RNA Viruses** | | | | |
| **Category** | **Morbidity** | **Year** | **Mortality** (if different from morbidity source) | **Year** |
| RSV | http://www.who.int/vaccine_research/diseases/ari/en/index2.html | 2008 |  |  |
| Measles | http://www.who.int/vaccine_research/diseases/ari/en/ | 2007 |  |  |
| Mumps | Weekly epidemiological record No. 48, 2005, 80, 417-424 | 2004 | Weekly epidemiological record No. 7, 2007, 82, 49-60. "Mumps virus vaccines" | 2004 |
| Rabies | http://www.who.int/rabies/epidemiology/Rabiessurveillance.pdf | 2005 |  |  |
| Ebola | http://www.who.int/mediacentre/factsheets/fs103/en | 2008 |  |  |
| Influenza (A,B,C) | http://www.who.int/vaccine_research/diseases/ari/en/index1.html | 2008 |  |  |
| **(+) sense, SS RNA Viruses** | | | | |
| Hepatitis C Virus | http://www.who.int/mediacentre/factsheets/fs164/en | 2000 | www.who.int/healthinfo/global_burden_disease/estimates_regional/en/index.html (Mortality and Morbidity-Deaths-WHO Regions[xls]) | 2004 |
| Dengue Virus | http://www.who.int/mediacentre/factsheets/fs117/en | 2007 | www.who.int/csr/disease/dengue/impact/en/ (WHO: Impact of Dengue) | 2008 |
| West Nile virus | Animal Health Res Rev 2008;9:71-86. [PMID 18348742] | 2007 |  |  |
| Yellow fever | http://www.who.int/mediacentre/factsheets/fs100/en/ | 2001 |  |  |
| Poliovirus | http://www.polioeradication.org/casecount.asp | 2006 |  |  |
| **Retro-Transcribing Viruses** | | | | |
| Hepatitis B Virus | www.who.int/mediacentre/factsheets/fs204/en/ | 2008 | Best Pract Res Clin Gastroenterol 2008;22:991-1008. [PMID:19187863]. |  |
| **Double Strand DNA Viruses** | | | | |
| Human herpesvirus 2 | www.scielosp.org/pdf/bwho/v86n10/a17v8610.pdf | 2003 | NA |  |
| Human Papillomavirus | www.who.int/mediacentre/factsheets/fs289/en/ | 2006 |  |  |
| **Actinobacteria/Proteobacteria** | | | | |
| Tuberculosis (Latent) | http://www.who.int/mediacentre/factsheets/fs104/en/ | 2005 |  |  |
| Tuberculosis (Active) | http://www.who.int/tb/publications/global_report/2008/key_points/en/ | 2006 |  |  |
| Leprosy | http://www.who.int/lep/en/ | 2007 | www.who.int/healthinfo/global_burden_disease/estimates_regional/en/index.html (Mortality and Morbidity-Deaths-WHO Regions[xls]) | 2004 |
| Vibrio cholerae | http://www.who.int/mediacentre/factsheets/fs107/en/ | 2006 |  |  |
| Haemophilus | http://www.who.int/mediacentre/factsheets/fs294/en/ | 2005 |  |  |
| Neisseria meningitidis | http://www.who.int/mediacentre/factsheets/fs289/en/ | 2006 | www.who.int/vaccine_research/diseases/soa_bacterial/en/index2.html (WHO: Bacterial Infections) | 2006 |
| Neisseria gonorrhoeae | http://www.who.int/vaccine_research/diseases/soa_std/en/index2.html | 2008 | www.who.int/healthinfo/global_burden_disease/estimates_regional/en/index.html (Mortality and Morbidity-Deaths-WHO Regions[xls]) | 2004 |
| Bordetella pertussis | http://www.who.int/immunization_monitoring/diseases/pertussis_surveillance/en/ | 2007 |  |  |
| **Firmicutes and Other Bacteria** | | | | |
| Group A Streptococcus | www.who.int/vaccine_research/diseases/soa_bacterial/en/index5.html | 2005 |  |  |
| Chlamydia trachomatis | /www.who.int/vaccine_research/diseases/soa_std/en/index1.html | 2008 | www.who.int/healthinfo/global_burden_disease/estimates_regional/en/index.html (Mortality and Morbidity-Deaths-WHO Regions[xls]) | 2004 |
| Helicobacter pylori | www.who.int/vaccine_research/diseases/soa_bacterial/en/ | 2008 | www.healthline.com/dictionary/Helicobacter pylori | 2005 |
| **Eukaryotes** | | | | |
| Schistosoma | The Lancet Infectious Diseases. 2006; 6(7) 411-25. “Schistosomiasis and water resources development: systematic review, meta-analysis, and estimates of people at risk.” Webappendix 2. | 2003 | www.who.int/healthinfo/global_burden_disease/estimates_regional/en/index.html (Mortality and Morbidity-Deaths-WHO Regions[xls]) | 2004 |
| Malaria | http://www.who.int/malaria/wmr2008/MAL2008-Chap3-EN.pdf | 2006 |  |  |
| Trypanosoma cruzi | Circulation. 2007; 116: 1876-1878. “Establishing Pediatric Cardiovascular Services in the Developing World A Wake-Up Call.” | 2007 | www.who.int/healthinfo/global_burden_disease/estimates_regional/en/index.html (Mortality and Morbidity-Deaths-WHO Regions[xls]) | 2004 |
| Leishmania | http://www.who.int/leishmaniasis/burden/magnitude/burden_magnitude/en | 2008 | www.who.int/healthinfo/global_burden_disease/estimates_regional/en/index.html (Mortality and Morbidity-Deaths-WHO Regions[xls]) | 2004 |
| **Cancer** | J ClinOncol 2006;24: 2137-2150. [PMID: 16682732] | 2002 | www.who.int/mediacentre/factsheets/fs297/en/ (WHO: Cancer) | 2007 |
| **HIV** | www.searo.who.int/LinkFiles/CDS_Factsheet_WAD2008.pdf | 2008 | www.avert.org/worldstats.htm (Worldwide HIV & AIDS Statistics) | 2007 |

Table S5: Worldwide morbidity and mortality figures were obtained, in large part, from the WHO using their publically available data. The WHO draws on a wide range of data sources to quantify global and regional effects of diseases, the details of which are not discussed here, however are available on their websites. When statistics were not available through the WHO, we used the peer-reviewed literature and/or personal communication with experts in the respective fields. Morbidity and mortality data for individual pathogens/diseases are acquired at different yearly intervals, data derived from a single year could not be determined for all of the pathogens included herein. The majority of the figures were available for years 2004-2008, however, some figures were older than 2004 and have been noted.
