# Supplementary material for: Classification of the Universe of Immune Epitope Literature: Representation and Knowledge Gaps
Source: PLoS One. 2009 Sep 14;4(9):e6948. doi: 10.1371/journal.pone.0006948 (PMC2747625; doi:10.1371/journal.pone.0006948)
Supplement: Table S6 — Worldwide morbidity and mortality figures were obtained, in large part, from the WHO using their publically available data. The WHO draws on a wide range of data sources to quantify global and regional effects of diseases, the details of which are not discussed here, however are available on their websites. When statistics were not available through the WHO, we used the peer-reviewed literature and/or personal communication with experts in the respective fields. Morbidity and mortality data for individual pathogens/diseases are acquired at different yearly intervals, data derived from a single year could not be determined for all of the pathogens included herein. The majority of the figures were available for years 2004–2008, however, some figures were older than 2004 and have been noted. (0.04 MB DOC) [file pone.0006948.s006.doc]

| **Table S6. Autoimmune Morbidity and Mortality Source** | | | | |
| --- | --- | --- | --- | --- |
| **Category** | **Morbidity** | **Year** | **Mortality** (if different from Morbidity source) | **Year** |
| **Type I Diabetes** | www.who.int/mediacentre/factsheets/fs312/en | 2008 | Not available |  |
| **Rheumatoid Arthritis** | Global Burden of Disease Report, 2004 | 2004 |  |  |
| **Multiple Sclerosis** | Atlas Multiple Sclerosis Resources in the World 2008, WHO | 2008 | Neurological disorders: Public health challenges, Annex 1, Table A.4.3. (www.who.int/mental_health/neurology/neurological_disorders_report_web.pdf) | 2005 |
| **Lupus** | Lupus Foundation of America  ‘World Lupus Day Observed’ May 1, 2008. | 2008 | Arthritis and Rheumatism 2006;54:2550-2557. | 2001 |
| **Myasthenia Gravis** | Expert Rev Neurother 2009;9;351-358. | 2009 | www.nationmaster.com/graph/mor_mya_gra_and_oth_myo_dis-myasthenia-gravis-other-myoneural-disorders_percap | 2004 |
| **Alzheimer's** | Global Burden of Disease Report, 2004 | 2004 |  |  |
| **Table 10. Autoimmune Morbidity and Mortality Source** | | | | |
| **Category** | **Morbidity** | **Year** | **Mortality** (if different from Morbidity source) | **Year** |
| **Type I Diabetes** | www.who.int/mediacentre/factsheets/fs312/en | 2008 | Not available |  |
| **Rheumatoid Arthritis** | Global Burden of Disease Report, 2004 | 2004 |  |  |
| **Multiple Sclerosis** | Atlas Multiple Sclerosis Resources in the World 2008, WHO | 2008 | Neurological disorders: Public health challenges, Annex 1, Table A.4.3. (www.who.int/mental_health/neurology/neurological_disorders_report_web.pdf) | 2005 |
| **Lupus** | Lupus Foundation of America  ‘World Lupus Day Observed’ May 1, 2008. | 2008 | Arthritis and Rheumatism 2006;54:2550-2557. | 2001 |
| **Myasthenia Gravis** | Expert Rev Neurother 2009;9;351-358. | 2009 | www.nationmaster.com/graph/mor_mya_gra_and_oth_myo_dis-myasthenia-gravis-other-myoneural-disorders_percap | 2004 |
| **Alzheimer's** | Global Burden of Disease Report, 2004 | 2004 |  |  |

Table S6: Worldwide morbidity and mortality figures were obtained, in large part, from the WHO using their publically available data. The WHO draws on a wide range of data sources to quantify global and regional effects of diseases, the details of which are not discussed here, however are available on their websites. When statistics were not available through the WHO, we used the peer-reviewed literature and/or personal communication with experts in the respective fields. Morbidity and mortality data for individual pathogens/diseases are acquired at different yearly intervals, data derived from a single year could not be determined for all of the pathogens included herein. The majority of the figures were available for years 2004-2008, however, some figures were older than 2004 and have been noted.
